# The Bacteriology and Its Virulence Factors in Neonatal Infections: Threats to Child Survival Strategies

**DOI:** 10.1155/2018/4801247

**Published:** 2018-07-02

**Authors:** Obiora Shedrach Ejiofor, Onyinye Mercy Ajunwa, Chijioke Elias Ezeudu, George Ogonna Emechebe, Kenneth Nchekwube Okeke, Christian Chukwuemeka Ifezulike, Ifeoma Mercy Ekejindu, Jude Nnaemeka Okoyeh, Eunice Ogonna Osuala, Angus Nnamdi Oli

**Affiliations:** ^1^Department of Pediatrics, Chukwuemeka Odumegwu Ojukwu University, Awka, Anambra State, Nigeria; ^2^Department of Pharmaceutical Microbiology and Biotechnology, Faculty of Pharmaceutical Sciences, Agulu, Nnamdi Azikiwe University, Anambra State, Nigeria; ^3^Department of Pediatrics, College of Health Sciences, Faculty of Medicine, Nnamdi Azikiwe University, Nnewi Campus, Anambra State, Nigeria; ^4^Department of Medical Laboratory Science, Faculty of Health Science and Technology, Nnamdi Azikiwe University, Nnewi Campus, Anambra State, Nigeria; ^5^Department of Clinical Laboratory Science, School of Health Sciences, Winston-Salem State University, Winston-Salem, NC, USA; ^6^Department of Nursing Sciences, Faculty of Health Science and Technology, Nnamdi Azikiwe University, Nnewi Campus, Nigeria

## Abstract

**Background:**

Neonatal infection refers to the infection of the newborn during the first twenty-eight days of life. It is one of the causes of infant morbidity and mortality worldwide. The aim of the study is to determine the relative contribution of the different pathogens to the overall disease burden. It will also determine the mechanisms of virulence of these pathogens that cause neonatal infections at Chukwuemeka Odumegwu Ojukwu University Teaching Hospital (COOUTH), Awka.

**Methods:**

Biological samples were collected from 30 neonates admitted at the special care baby unit (SCBU) of COOUTH and cultured using selective media and nutrient agar. The isolates were identified using microbiological and biochemical tests. The antibiogram study was determined using Kirby-Bauer disc diffusion method on Mueller Hinton Agar. Several methods previously reported in literature were used for the characterization of the virulence factors.

**Results:**

From the 30 blood samples collected,* Pseudomonas* spp. (19.7%),* Escherichia coli* (23%),* Salmonella* spp. (24.6%), and* Staphylococcus aureus* (32.8%) were isolated. Male to female ratio of study population was 1.5: 1. The isolates were 100 % resistant to ticarcillin, cephalothin, ceftazidime, and cefuroxime but appreciably susceptible to only levofloxacin (88.85%). They were moderately susceptible to ceftriaxone/sulbactam (39.05%) and azithromycin (26.46%). Common virulence factors identified among the isolates (up to 90 %) were hemolysin, biofilm formation, and acid resistance. Less common virulence factors were proteases (50 %), deoxyribonucleases (50 %), enterotoxins (63%), and lipopolysaccharide (70%). The virulence factors were found mostly among the* S. aureus* isolates.

**Conclusions:**

* Pseudomonas* spp.,* Escherichia coli*,* Salmonella* spp., and* Staphylococcus aureus *were implicated in neonatal infections in the center and most of them were resistant to conventional antibiotics. The organisms showed marked virulence and multidrug resistance properties. Levofloxacin, a fluoroquinolone, had superior activity on the isolates compared to other antibiotics used in the study.

## 1. Background

Neonatal infections or sepsis refers to the infections of the newborn during the neonatal period (the first twenty-eight days of life). A positive blood culture in the first four weeks of life is generally taken as being diagnostic of the infection [1]. Neonatal sepsis has been divided into two types based on the time of onset: namely, early onset sepsis occurring within the first 72 hours of birth and late onset which occurs after 72 hours to 28 days. Early-onset is usually associated with acquisition of microorganisms from the mother, while late-onset sepsis is caused by bacteria from the healthcare environment [2, 3]. At delivery, maternal genitourinary tract colonization by pathogens may precipitate transplacental infection and/or an ascending infection from the cervix as the neonate passes through the colonized birth canal [4]. The infection is one of the four leading causes of neonatal morbidity and mortality worldwide accounting for 23.4% of 3.072 million neonatal deaths globally [1, 5, 6]. It continues to be a major problem for neonates in intensive care units around the world [2, 7]. In developing country, neonatal mortality and stillbirth remain significantly high when compared to the developed countries [5, 8]. In Nigeria, neonatal mortality accounts for about two-thirds of infant mortality [9].

A variety of Gram-positive organisms such as* Streptococcus* and* Staphylococcus aureus* as well as Gram-negative bacteria like* Escherichia coli* have been implicated [10, 11]. Fungal and protozoan organisms have also been identified as causative agents in neonatal sepsis [5]. These organisms exhibit a wide range of susceptibility to different classes of antibiotics.

Over time, there has been significant variation and changes in the spectrum of the organisms implicated in neonatal sepsis. Importantly, the susceptibility of these organisms to a spectrum of antibiotics has equally changed with time. This is mainly due to irrational use of antibiotics and use of fake and substandard drugs [12]. The emergence of antibiotic resistance in the management of infectious diseases constitutes a major public health problem especially in the developing countries [13, 14]. There is need for periodic evaluation of organisms that cause neonatal sepsis so as to improve its management. It is also important to understand the mechanism of virulence of the organisms causing such infections. To the best of the knowledge of the authors, there is a gap in the knowledge of the pathogens, and their virulence factors, implicated in neonatal sepsis in Southeast Nigeria. This study is an attempt to fill this gap. The study outcome will assist evidence-based decisions for effective management and control of neonatal infections and will enhance child survival.

## 2. Methods

### 2.1. Study Area and Population

The study was carried out between April and August 2016 in the special baby care unit of Chukwuemeka Odumegwu Ojukwu University Teaching Hospital, Amaku of Awka in Anambra State of Nigeria. It is a public hospital owned by the State government and situated in the State Capital. It serves as a referral hospital, serving several neighboring cities. It also serves for the training of medical students, house officers, doctors undergoing residency training, nursing students, and pupil pharmacists. The center was chosen for the study based on its position as a referral and training center and is also strategic in child welfare in the State.

### 2.2. Selection Criteria

Clinically septic neonates (n=30) on admission in the special baby care unit of the hospital and whose mothers had not used any antibiotic 7 days before recruitment and sample collection were evaluated. Informed consent of the mother/legal caregivers of the baby was obtained.

### 2.3. Sample Collection

Thirty peripheral blood samples were used for the study. Out of the 11 neonates that presented within 24 hrs of birth, 10 also had their umbilical cords swabbed and cultured (the umbilical cord of the remaining neonate could not be reached and so was not swabbed). Patients' recruitment and samples collection were done by the pediatricians. Samples were transported immediately to the microbiology laboratory of the Department of Pharmaceutical Microbiology and Biotechnology, Faculty of Pharmaceutical Sciences, Agulu, Nnamdi Azikiwe University, Awka, for analysis. Each specimen was appropriately labeled and analyzed within an hour of collection. Prior to sample collection, sociodemographic data were obtained from the mothers/caregivers of the neonates. The study was conducted after obtaining due ethical approval (Approval Number: COOUTH/AA/VOL.1.005) from the hospital's ethics board.

## 3. Microbiological Analysis

### 3.1. Sample Processing

The umbilical cord swab and peripheral blood samples were separately and aseptically inoculated onto previously prepared and labeled sterile nutrient broth in test tubes. Thereafter, the tubes were covered properly using sterile cotton wool. The test tubes were incubated for 24 h and turbidity in any of the test tubes containing the inoculum was recorded as growth. Each inoculum that had growth was aseptically inoculated onto previously prepared and sterilized MacConkey agar plates, Mannitol salt agar plates, Salmonella-Shigella agar, and Cetrimide agar plates (i.e., each sample was in triplicate; one sample per the three media). The inoculated agar plates were incubated aerobically at 37°C for 24 h. The growths were inspected to identify the bacteria colonies and recorded.

### 3.2. Subculture of Isolated Colonies

To obtain pure isolates, discrete colonies of pathogens isolated were subcultured by inoculating onto sterile nutrient agar plates. The plates were incubated aerobically at 37°C for 24 h. After the incubation, colonies were subjected to microscopic examination and appropriate biochemical tests for identification. The pure isolates were also transferred aseptically into nutrient agar slants for future analysis/use.

### 3.3. Identification and Characterization of Isolates

Identification of bacterial isolates was done on the basis of their cultural, microscopic, and biochemical characteristics. The various isolates obtained were characterized by subjecting them to Gram staining and morphological (colonial morphology) tests using the standard methods.

### 3.4. Biochemical Tests

Specific biochemical tests were carried out to further identify the organisms and they include catalase test, coagulase test, indole test, and oxidase test.

### 3.5. Catalase Test

This was done on all Gram-positive cocci to differentiate* Staphylococcus* species from* Streptococcus* species. Using a sterile syringe, 2 drops of hydrogen peroxide (H_2_O_2_) were placed on a clean glass slide. A sterile cooled wire loop was used to collect a small portion of the test organism, placed in the drop of H_2_O_2_ on the slide and emulsified. Positive result gave effervescence, while negative result gave no effervescence.

### 3.6. Oxidase Test

This was done on the Gram-negative bacilli to identify* Pseudomonas* species among other Gram-negative bacilli. Two drops of oxidase reagent were placed on a piece of filter paper on a slide. With the edge of another slide, a good colony of the test organism was collected and smeared on the emulsified filter paper. A positive reaction turned the paper dark purple within 10-20 seconds, while a negative reaction shows no immediate color change.

### 3.7. Indole Test

This was done on all Gram-negative bacilli for the identification of* Enterobacteriaceae*:* Escherichia coli, Proteus *sp., and* Klebsiella* spp. The test organism was inoculated in a test tube containing 3 mL of sterile tryptone water and incubated at 37°C for 48 h. 0.5 mL of Kovac's reagent was added and shaken gently. The product was examined for a red coloration in the surface layer within 10 mins. A positive result gave red surface layer, while a negative result gave no red surface layer.

### 3.8. Sugar Fermentation Test

Sugar fermentation test was used to determine the bacterial isolates that could utilize different sugars as carbon source with the production of acid or gas. A 1% sugar medium was used. This was done by dissolving 1g of various sugars in 99ml of prepared peptone water contained in already labeled different beakers. The sugar medium was heated to active homogenous mixture. A total of 30 drops of bromothymol blue indicator was added to the media and shaken gently. 10ml was dispensed in test tubes containing inserted Durham tubes. The tubes were capped packed in bigger test tubes and loaded in autoclave and sterilized at 110°C for 10 minutes. After sterilization, it was allowed to cool and test organisms were inoculated into the tubes as labeled. The culture was incubated at 37°C for 24 to 48 h. The culture was observed for color change and gas production. Tubes that turned yellow was taken as acid positive and Durham tubes that had bubbles in them were recorded as gas positive.

## 4. Virulence Tests for the Isolates

Several factors of microbial origin are responsible for bacterial virulence properties. A few of these factors were tested to identify the ones possessed by the bacteria implicated in neonatal infections in the study site. The virulence tests performed on the isolates were antibiotic susceptibility analysis, enzyme analysis, acid resistance (pH 3.5) tests, toxins production tests, lipopolysaccharide test, haemoglobin utilization test, and biofilm formation test.

### 4.1. Antibiotic Susceptibility Testing

Antibiotic susceptibility of pure cultures of confirmed isolates was performed using Mueller Hinton Agar, by the standard Kirby-Bauer disc diffusion method. Standardized overnight culture of each isolate was prepared by inoculating each isolate into 3 mL sterile nutrient broths in test tubes and incubating at 37°C for 24 h and was made to match with 0.5 McFarland turbidity standards (equivalent to 1.5×10^8^ CFU/ml). The Mueller Hinton Agar used was prepared based on the manufacturer's specification. It was sterilized by autoclaving, poured into sterile Petri dishes, and allowed to solidify. These standard inoculums were swabbed under aseptic conditions, on the solidified Mueller Hinton Agar plates, and were allowed to soak for about 5 minutes. After this, the antibiotic disks were aseptically placed on the surface of the media and pressed down gently to lap with the media. The plates were incubated at 37°C for 24 h. At the end of 24 h, the inhibition zones created by each antibiotic against each isolate were measured and recorded as Inhibition Zone Diameter (IZD). This was performed in duplicate for each antibiotic per isolate and the average was obtained. Susceptibility/resistance was interpreted according to Clinical and Laboratory Standard Institute (CLSI) guidelines [15].

All the isolates showed multiple antibiotic resistances and were further subjected to other virulence tests as follows.

### 4.2. Enzyme Analysis

#### 4.2.1. Coagulase Test

The slide method was used. A drop of distilled water was placed on a clean glass slide using a sterile syringe. A flame sterilized wire loop was to pick a colony of the test microbial isolate from the culture plate and emulsified in the drop of distilled water on the slide. Later, a drop of undiluted plasma was added to it and mixed by tilting the slide to and fro while watching out for coagulation. A positive result showed immediate clumping within 10 seconds, while a negative result showed no clumping.

#### 4.2.2. Hyaluronidase

This test was conducted according to Edberg et al. [16] method. Briefly, nutrient agar to which hyaluronic acid (the substrate) from human umbilical cord (Sigma) had been added was inoculated with 24 h culture colonies and incubated at 37°C for 48 h. Then, the culture plate was flooded with 2N acetic acid and observed for 10 min. A zone of hydrolysis (clear zone) around the colonies shows the enzyme production by the bacteria colonies.

#### 4.2.3. Protease

This was according to Suganthi et al. [17] method. Skim milk agar supplemented with 5% NaCl and 1% casein was inoculated with 18 h pure bacteria culture and incubated overnight at 37°C for 24 hr. A clear zone, resulting from casein hydrolysis, seen around the inoculum spot was taken as a proof of enzyme production.

#### 4.2.4. Lipase

A loopful of 18 h pure bacteria colonies was streaked onto tributyrin agar plate and incubated at 37°C for 24 h and then observed for zone of hydrolysis around the colony. The appearance of a clear halo around the inoculum spot is indicative of lipase production [18].

#### 4.2.5. Deoxyribonuclease (DNase)

The method described by Pimenta et al. [19] was used. Briefly, DNase agar (containing toluidine blue and methyl green) plates were inoculated by spotting a loopful of overnight pure cultures on to the surface of the media, followed by aerobic incubation at 37°C for 48h. The plates were then flooded with 0.1 % 1 N HCl. The development of a red color or a zone of clearing indicated a positive result.

#### 4.2.6. Haemolysin

Pure cultures of the bacterial isolates were grown on the surface of 5 % defibrinated sheep blood agar in a trypticase soy agar base (Difco) and incubated at 37°C for 72 h. Lysing of the red blood cells indicated by a clear halo around the inoculum spot is indicative of haemolysin production [16].

#### 4.2.7. Detection of Superoxide Dismutase (SOD) Activity

Superoxide dismutases (SODs) catalyze the dismutation reaction (conversion) of superoxide anion (O_2_^−^) to hydrogen peroxide and molecular oxygen. Autoclaved yeast extract broth (10ml) was exposed to fluorescent light (15W for 3h) for the purpose of generating superoxide radicals and hydrogen peroxide. This was mixed with nutrient agar supplemented with water‐soluble, yellow‐colored nitro blue tetrazolium (0.1mg/ml), reautoclaved, and allowed to solidify. A 24 hr bacterial culture was inoculated onto the agar and incubated aerobically at 37°C for 48h. SOD activity was assessed by measuring the inhibition of the reduction of nitro blue tetrazolium (NBT) by superoxide free radical (O_2_^−^). The presence of clear zones (achromatic zones) around the inocula spots indicates SOD activity [20].

### 4.3. Antiphagocytic Factor (Fibrinolysin)

As described by Edberg et al. [16], fibrinogen (Sigma) was mixed with nutrient agar to yield a final concentration of 280 mg per 100 ml. Colonies were inoculated onto the surface of the agar and incubated at 37°C for 48 h. Clear zones around the inoculum spot with diameters > 2 mm indicate the presence of the clumping-factor (fibrinolysin) that impairs phagocytosis (a positive result).

### 4.4. Enterotoxins Production

Enterotoxins are proteinaceous, low molecular weight, and water-soluble exotoxins released by microorganisms. They are either chromosomally or plasmid encoded and act by binding to the major histocompatibility complex proteins of the pathogen's hosts thereby causing increased chloride ion permeability in the apical membrane of the epithelial cells of the host intestinal wall resulting in cell death [21]. Enterotoxins production was verified using Coagglutination Test Reagent (*Staphylococcal* Coagglutination (CoA) test). This test was carried out to determine the enterotoxigenicity the* E. coli*,* Salmonella* spp., and* Staphylococcus aureus* isolates. The method used (with some modifications) has been described by [22, 23]. The isolates were cultured on blood agar for 24h. Thereafter, a loopful of the culture was suspended in 0.1 ml of saline containing Polymyxin B (2 mg/ml) and incubated at 37°C for 1h. Triton X-100 (0.1%) was then added and the mixture was incubated again for 10 min to produce cell lysates which were then centrifuged at 3,000 x g (20°C) for 20 min). Equal volumes of cell lysates (25 *μ*l) and Coagglutination (CoA) test reagent (formalin-treated and heat-killed cells of* Staphylococcus aureus* Cowan type-1 strain coated with a high-titer rabbit anti-LT (antiheat labile enterotoxin) serum) were mixed on a glass slide. Positive results show agglutination within 2 min.

### 4.5. Shiga Toxin Production

Culture samples were plated onto sorbitol MacConkey agar (SMAC) and incubated at 35°C for 24 h and then examined for non-sorbitol-fermenting colonies as described by Vallières et al. [24].

### 4.6. Acid Resistance (pH 3.5) Test

The method suggested by Edberg et al. [16] was used with some modifications. Briefly, using sterile dechlorinated water, a 1.0 McFarland turbidity equivalent of the test organisms was prepared and allowed to acclimatize for 24 h at room temperature. A previously titrated amount of 1N acetic acid was mixed with 1 ml of this suspension until a final pH of 3.5 is obtained. After rapid mixing, the suspension was incubated at room temperature for 10 min. 0.1 ml of the bacterial suspension was subcultured onto a sterile nutrient agar plate buffered with HEPES to a final pH of 7.5. This buffered agar plate neutralized the acetic acid while allowing the bacteria to grow. Percent survival was calculated using the equation

(1) $$\begin{array}{c} \%\;\;Survival=\frac{\left(\text{Final }number\text{ of CFU}/ml\;\;that\;\;survived\;\;on\;\;the\text{ buffered }agar\;\;plate\right)\text{100}}{Initial\;\;inoculum\;\;size\;\;obtained\text{ from }quantitative\;\;plating\text{ of }the\;\;suspension\text{ after }24\;\;h\;\;at\;\;room\;\;Temp.} \end{array}$$

### 4.7. Biofilm Formation

This was done using the tube adherence test suggested by Christensen et al. [25] with little modification. Briefly, Müeller-Hinton (MH) medium was inoculated with 24 h bacterial culture in test tubes and incubated aerobically at 37°C for 48 hrs. The contents of the test tubes were discarded and the tubes were stained with 0.1% safranine, washed with sterile water thrice, and dried. A positive result was taken as the presence of a layer of the stained material that adhered to the inner wall of the tubes. If only a stained ring was seen at the liquid-air interface, it was considered as no biofilm formation. Experiments were done in duplicate for three times and results were recorded as very strong, strong, weak, and absent.

### 4.8. Lipopolysaccharide/Endotoxins (1 Endotoxin Unit = 0.2 ng Lipopolysaccharide)

Clinical manifestations of Gram-negative bacterial infections are affected by how and where animal hosts sense the lipopolysaccharide (LPS) on the cell wall [26]. Silver-staining detection method developed by Kittelberger and Hilbink [27] was used. The lipopolysaccharides are stained dark brown by the silver stain.

### 4.9. Haemoglobin Utilization

Many pathogenic bacteria utilize haemin and haemoglobin as iron sources through processes linked to heme transport systems and outer membrane heme-binding proteins [28]. To establish if the organism utilizes haemoglobin as an iron source 5 % defibrinated sheep blood agar supplemented with 0.5% yeast extract, menadione (0.05 mg/ml) and 0.075% cysteine were used for the cultivation of the bacteria at 37°C for 48 h. A clearing around the inoculum spot is indicative of haemoglobin utilization.

### 4.10. Data Analysis

Data analyses were done with GraphPad Prism version 5.00 for Windows, GraphPad Software, Inc., San Diego, California, USA, www.graphpad.com. The statistical tests used were* t*-test, Chi square test, One-Way ANOVA with Dunnett's Multiple Comparison Test, and Two-Way ANOVA with Bonferroni posttests. All P values reported are for a two-tailed test. The significance level was taken as P < 0.05.

## 5. Results

**Figure 1:**
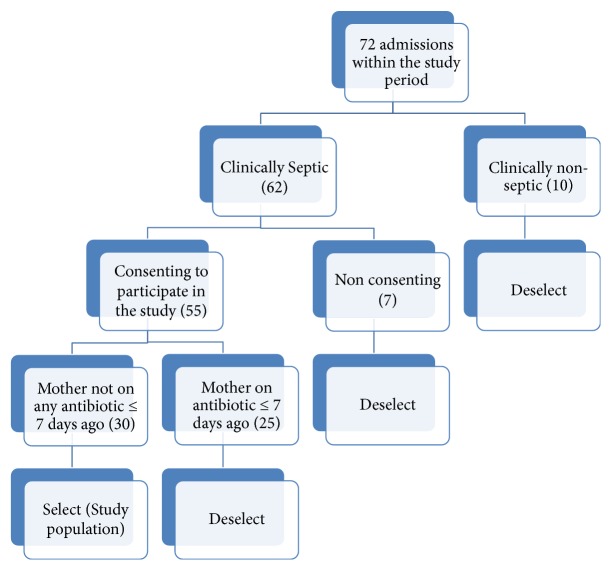
Selection criteria.

A total of 30 neonates (Figure 1), comprising 12 males and 18 females, admitted with clinical signs of sepsis at the special care baby unit of Chukwuemeka Odumegwu Ojukwu University Teaching Hospital, Awka, were assessed within the period under review.

**Figure 2:**
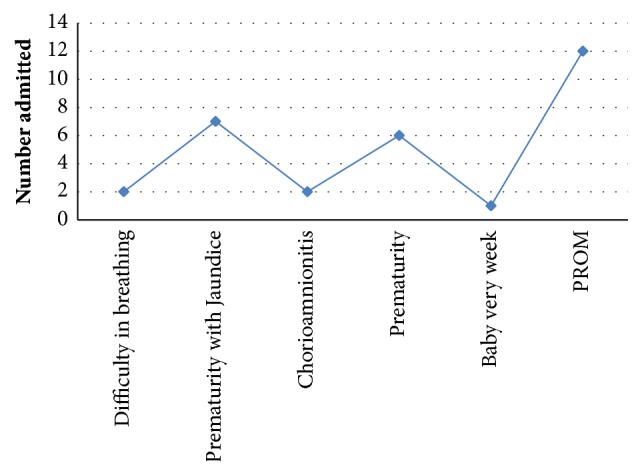
Clinical assessments of the selected babies (reasons for admission).

Most of the babies that met our selection criteria were born premature (Figure 2). These included prematurity with jaundice, prematurity, and premature and/or prolonged rupture of membranes (PROM), giving a total of 25 cases.

All the samples showed visible bacterial growth after 24h incubation (Table 1). The relative contributions of the different pathogenic bacteria to the overall disease burden were in the order* Pseudomonas* spp. (19.7%),* Escherichia coli* (23%),* Salmonella* spp. (24.6%), and* Staphylococcus aureus* (32.8%). However, there was no statistical difference in the occurrence of the isolates (p < 0.05) and no gender preponderance of neonatal sepsis (p valve = 0.3202). The prevalence of pathogens was higher in premature babies than in mature babies (p value = 0.0189). Babies born preterm were more likely to have infections than those born at term (p value < 0.05).

The neonatal age range was 10-398 hrs with a mean age of 93.2 hrs (Table 2). Of those with early-onset sepsis (total = 16), 11 (68.75%) presented within 24 hours and 3 (18.75%) presented at 24-48 hours, while 2 (12.5) presented within 48-72 hours. One-Way ANOVA showed that age had no effect on type of bacterial isolate (P value = 0.8474). It can be inferred, therefore, that neonatal infection can be caused by any of the isolates; age group and sex notwithstanding. All the isolates are therefore considered potentially virulent. However, neonatal age significantly (P value = 0.0299) affected the relative risk of being infected by microorganisms. Dunnett's Multiple Comparison Test showed significant difference at early-onset infections when compared with onsets > 216 hrs after delivery.

Table 3 shows that the fluoroquinolone antibiotic, levofloxacin, fared better than the rest of the drugs. Two-way ANOVA showed that the antibiotics used do not have the same effect on all the organisms. The interaction between the drugs and the organisms accounts for 9.42% of the total variance with a p value = 0.2811. The interaction is considered not significant. Also, the kind of antibiotic used affected the antimicrobial resistance pattern of the organisms. The kind of antibiotic used accounted for 76.92% of the total variance with a p value of < 0.0001. The effect is considered extremely significant. The organisms isolated did not affect the susceptibility to the antibiotics used as the organisms account for only 2.19% of the total variance with a p value = 0.0697. The effect is considered not quite significant. Bonferroni posttests revealed that levofloxacin significantly (p value <0.05) performed better than the rest of the antibiotics used in the study and also significantly affected (p value <0.05) most of the organisms isolated from the neonates. However, the effect on* E. coli* was not significantly better than the effect of ceftriaxone/sulbactam (p value > 0.05).

Table 4 showed that the most common virulence factors possessed by the isolates were hemolysin and biofilm formation and are acid resistant. Up to 90 % (27) of the isolates produced them. Other common virulence factors were proteases (50 %), DNases (50 %), enterotoxins (63%), and lipopolysaccharide (70%). The virulence factors were found mostly among the* S. aureus* isolates.

## 6. Discussion

In this study,* S. aureus* was the most implicated pathogen in neonatal infections during the study period. Several studies [29–31] had reported similar findings. The prevalence of pathogens causing neonatal infections was higher in preterm babies than in term babies. This is also confirmed in previous report [30]. Several factors such as underdeveloped innate immune system, prematurity, and invasive life-saving medical interventions could predispose these neonates to infection from their environment. In studies where mothers had preterm rupture of membranes (PROM), 14.1% of the newborns had clinical signs of infection, where mothers had preterm premature rupture of membranes (PPROM), and 32.1% of the newborns had clinical signs of infection [2, 4]. The present study showed that the prevalence of neonatal infection is higher among neonates born to mothers with preterm labor possibly because of their immature immune system.

This study also showed that pathogens were more implicated in peripheral blood than the umbilical cord swab samples. A contrary report was documented by other researchers where the authors discovered that a total of 24.4% high risk newborns had positive lab culture when umbilical cord blood sample was used (11 out of 45) and 8 out of 45 newborn had positive peripheral blood sample culture [32]. Differences in specimen samples could explain this disparity; whereas the present study also used umbilical cord swab as specimen, Kalathia et al. [32] used only umbilical cord blood as specimen. More so, the direct connection between the mother and the neonate through the umbilical cord makes the newborn more vulnerable to ascending infection from the mother. It is important to note that after delivery, the umbilical cord does not fall off completely and is exposed to external environment. This exposure can lead to neonatal acquisition of microorganisms from the immediate environment via the umbilical cord. The higher chance of early-onset sepsis as shown in this study supports previous reports [3, 7, 9].

S*. aureus*, Gram-positive organism, was the most predominant pathogen implicated in neonatal infections in this study. The high prevalence of Gram-positive bacteria in neonatal intensive care units had also been reported by other researchers [30, 31, 33]. Enteric bacilli from the digestive system of the mother (in this study,* Salmonella *spp. and* E coli*) were the second most prevalent microorganisms causing neonatal infections and had been reported to be one of the risk factors in very low birth weight infants [10, 34]. These microorganisms inhabit the intestinal tract of humans.

Antibiogram study showed that levofloxacin had the highest activity compared to other antibiotics used in this study. Levofloxacin, which is a fluoroquinolone, had been reported to have superior activity on organisms such as* E. coli, Staph aureus, Pseudomonas *spp.,* and Salmonella *spp. [35]. Beta-lactam antibiotics had the least activity. The implication is that these organisms may have become resistant to the beta-lactam antibiotics by producing beta-lactamase enzymes which breaks down the beta ring of the antibiotics. Ceftriaxone/sulbactam was the second most active antibiotic used in this study. The enhancement of the activity of ceftriaxone by sulbactam through the inhibition of the beta-lactamase degradation of ceftriaxone is well documented [36].

Virulence factors are biological molecules produced by pathogenic organisms that help them grow and survive in their hosts. These biomolecules assist and promote host colonization and may also cause damage to the host and eventual death of the host if interventions are not instituted. Some virulence factors like bacterial toxins are encoded on mobile genetic elements and are spread through horizontal gene transfer as seen in* Escherichia coli* O157:H7 [37–39]. The chaperone/usher (CU) pathway is responsible for the biogenesis of virulence-associated pili by many different Gram-negative bacteria [40, 41].

The high level of drug resistance seen among the bacterial isolates causing neonatal infection in the study might be linked to the possession of some virulence factors. In response to antibiotics exposure, bacteria usually activate regulatory systems responsible for controlling the expression of genes involved in the conversion of the drugs to harmless compounds [20]. Bacteria possessing DNase can escape from neutrophil extracellular traps (NETs), thereby helping them survive [42]. Hyaluronidases as essential virulence factors in pathogens destroy the polysaccharide that holds host cells together, thereby making it easier for the pathogen to spread and destroy the host organism [43]. Biofilm formation, proteases, and lipases are major virulence factors reported in pathogenic bacteria [44, 45]. Antiphagocytic factor (Fibrinolysin), hemolysins, and coagulase are important virulence factors also reported in pathogenic bacteria [46, 47]. Superoxide dismutase (SOD) contributes to the virulence of many human-pathogenic bacteria as it assists the pathogen to evade the actions of phagocytic cells [48] and “neutralize toxic levels of reactive oxygen species generated by the host” [49]. Lipopolysaccharide plays some role in adhesion of pathogens to host cells. Many pathogenic bacteria utilize haemin and haemoglobin as iron sources through processes linked to heme transport systems and outer membrane heme-binding proteins [28]. Fatty acids as a major component of host cell membranes provide both physical and chemical barrier that insulates intracellular reactions from environmental fluctuations and in some cases regulate stress resistance and pathogen's virulence factor production [50]. Pathogens that can resist the actions of these acids have resistance against phagosomal killing in macrophages [50].

## 7. Conclusions

The study showed that* Pseudomonas* spp.,* Escherichia coli*,* Salmonella* spp., and* Staphylococcus aureus* were the key pathogens implicated in neonatal infections in the center with most being resistant to conventional antibiotics and possessing virulence factors.* Staphylococcus aureus* contributed most to the overall disease burden. Levofloxacin, a fluoroquinolone, had superior activity against the key pathogens compared to other antibiotics used in the study.

Therefore, it is suggested that health professionals should enlighten mothers on the measures to be taken to prevent neonatal infections. Such measures may include proper antenatal care, good hygiene, and avoidance drug abuse. Health professionals and hospital management should also implement measures that reduce nosocomial infections.

## Figures and Tables

**Table 1:** The prevalence of the isolates in relation to gender and maturity of the neonates.

**Organisms**	**Percentage prevalence**	**Gender of the Neonates**			**Total**	**Neonate delivered**		
		**X** ^**2**^	**Male**	**Female**		**Preterm**	**Full-term**	***t* test**
***E. coli***	23.00	**p valve = 0.3202**	7	5	**12**	10	2	**p valve = 0.0189**
***Salmonella* spp**	24.6		2	1	**3**	3	0	
***Pseudomonas *spp**	19.7		5	4	**9**	7	2	
***S. aureus***	32.8		4	2	**6**	5	1	

**Total**			**18**	**12**	**30**	**25**	**5**	

**Table 2:** Frequency of occurrence of the isolates.

**Organisms**	**Age**_ _^**∗∗∗**^ ** (hrs)**				
	**≤ 72**	**73 - 216**	**217 - 288**	**> 288**	
***E. coli***	8	1	1	0	**P value = 0.8474**
***Salmonella* spp**	3	2	0	0	
***Pseudomonas *spp**	4	4	0	1	
***S. aureus***	1	3	1	1	

**Total**	**16**	**10**	**2**	**2**	
**Age Interval**	**62**	**144**	**72**	**110**	
	**P value = 0.0299**				

**Table 3:** The multidrugs resistance pattern of the isolates.

**Organisms**	%** Resistance of the isolates to antibiotics**																				**ANOVA**
	**TIC (100)**		**KF (100)**		**CAZ (100)**		**C/S (60.95)**		**MEM (87.91)**		**LVD (11.15)**		**CXM (100)**		**PRL (100)**		**HFX (87.91)**		**HVX (73.54)**		
	**B**	**UC**	**B**	**UC**	**B**	**UC**	**B**	**UC**	**B**	**UC**	**B**	**UC**	**B**	**UC**	**B**	**UC**	**B**	**UC**	**B**	**UC**	
***S. aureus***	100	100	100	100	100	100	41.7	75	100	100	0	25	100	100	100	100	100	100	100	37.5	
***E. coli***	100	100	100	100	100	100	33.4	12.5	83.3	87.5	16.7	12.5	100	100	100	100	33.3	100	33.3	75	
***Pseudomonas***	100	100	100	100	100	100	100	75	80	87.5	0	25	100	100	100	100	80	100	80	62.5	
***Salmonella***	100	100	100	100	100	100	50	100	75	90	0	10	100	100	100	100	100	90	100	100	
**ANOVA **																					

KEY:

UC: umbilical cord swab samples and B: peripheral blood samples. Figures in brackets represent mean resistance of the isolates to the drugs tested. TIC: ticarcillin 75*μ*g, KF: cephalothin 30*μ*g, CAZ: ceftazidime 30 *μ*g, C/S: ceftriaxone/sulbactam 30 *μ*g, MEM: meropenem 10*μ*g, LVD: levofloxacin 5 *μ*g, CXM: cefuroxime 30 *μ*g, PRL: piperacillin 110 *μ*g, HVX: azithromycin 15 *μ*g, and HFX: ceftriaxone 30 *μ*g.

**Table 4:** Virulence factors characterization of the isolates.

**Virulence Factors**		**Bacteria (**%**)**				**Total**
		*E. coli* n = 12	*Salmonella *spp n = 3	*Pseudomonas *spp n = 9	*S. aureus* n= 6	
**Enzymes**	Hyaluronidase	0	0	8	6	**14**
	Proteases	0	0	9	6	**15**
	Lipases	0	0	9	5	**14**
	DNases	0	0	9	6	**15**
	Hemolysins	12	3	7	5	**27**
	Coagulase	0	0	0	6	**6**
	Superoxide dismutase	9	3	0	0	**12**
	Fibrinolysin	0	0	2	6	**8**

**Toxins and others**	Enterotoxins	10	3	0	6	**19**
	Shiga toxin	12	0	0	0	**12**
	Acid resistance pH 3.5	10	3	8	6	**27**
	Biofilm formation	10	3	8	6	**27**
	Lipopolysaccharide	12	1	8	0	**21**
	Haemoglobin utilization	12	0	0	0	**12**

	**Total**	**87 (52**%**)**	**16 (38**%**)**	**68 (54**%**)**	**58 (69**%**)**	

## Data Availability

The data used to support the findings of this study are included within the article.
